# Analyzing the Role of Receptor Internalization in the Regulation of Melanin-Concentrating Hormone Signaling

**DOI:** 10.1155/2013/143052

**Published:** 2013-11-17

**Authors:** Jay I. Moden, Katrina Haude, Robert Carroll, Andrew Goodspeed, Laurie B. Cook

**Affiliations:** Department of Biology, 217 Lennon Hall, The College at Brockport, State University of New York, 350 New Campus Drive, Brockport, NY 14420, USA

## Abstract

The regulation of appetite is complex, though our understanding of the process is improving. The potential role for the melanin-concentrating hormone (MCH) signaling pathway in the treatment of obesity is being explored by many. It was hypothesized that internalization of MCH receptors would act to potently desensitize cells to MCH. Despite potent desensitization of ERK signaling by MCH in BHK-570 cells, we were unable to observe MCH-mediated internalization of MCH receptor 1 (MCHR1) by fluorescence microscopy. A more quantitative approach using a cell-based ELISA indicated only 15% of receptors internalized, which is much lower than that reported in the literature. When *β*-arrestins were overexpressed in our system, removal of receptors from the cell surface was facilitated and signaling to a leptin promoter was diminished, suggesting that internalization of MCHR1 is sensitive to cellular *β*-arrestin levels. A dominant-negative GRK construct completely inhibited loss of receptors from the cell surface in response to MCH, suggesting that the internalization observed is phosphorylation-dependent. Since desensitization of MCH-mediated ERK signaling did not correlate with significant loss of MCHR1 from the cell surface, we hypothesize that in this model system regulation of MCH signaling may be the result of segregation of receptors from signaling components at the plasma membrane.

## 1. Introduction

Obesity results when caloric intake exceeds metabolic needs over an extended period of time. The condition predicates heart disease and diabetes—two pathologies that diminish the quality of life and increase risk of premature death. While appetite control is incredibly complex, we do know that a hormone discovered in teleost fish, melanin-concentrating hormone (MCH), plays a role in regulating feeding behavior in higher-order mammals [1–5]. MCH acts via a G protein-coupled receptor (GPCR) at the plasma membrane of many cell types including neurons to stimulate appetite and adipocytes to stimulate the synthesis and secretion of leptin. Studies with MCHR1 knockout mice have shown similar physiological adaptations including an increase in resistance to diet-induced obesity and hyperphagia compared to wild-type mice fed a similar diet [6]. Recently, loss of MCHR1 localization to primary cilia has been linked to the severe obesity seen with Bardet-Biedl syndrome [7], suggesting that regulation of MCH signaling plays an important role in energy homeostasis and that this pathway could become a pharmacological target to curb appetite. The usefulness of an MCHR1 antagonist in treating human obesity is being explored by others; an Early Phase I Clinical Trial has seen promising results [8].

The dramatic phenotype observed in loss of function studies suggests that irregular MCH signaling patterns have the potential to cause disease. Unless appetite signaling is properly desensitized following food consumption, hunger may continue. The desensitization of MCHR1 was first described by Lembo et al., who observed internalization of MCHR1 by fluorescence microscopy following agonist treatment [9]. This was supported by evidence that MCHR1 internalization occurs via the canonical *β*-arrestin and clathrin-mediated pathway [10, 11]. Flag-tagged MCHR1 internalized in HEK293T cells following 30 min MCH treatment to only 44.2% of initial surface receptor levels [11]. Further studies suggested that *β*-arrestin 2 is preferentially recruited to MCHR1; however, they were performed in the presence of overexpressed GRK2, a kinase that increases the affinity of GPCRs for arrestins [10]. 

The goal of this study was to further characterize the agonist-mediated desensitization of MCHR1. We unexpectedly discovered early on that despite rapid and extensive desensitization of ERK activation in response to MCH, agonist-mediated internalization of MCHR1 was very poor in our model, which is contrary to what others have reported [10, 11]. Although we were able to significantly improve receptor internalization by overexpression of *β*-arrestins or GRK2, this study suggests that there is an unexplored desensitization mechanism that occurs at the plasma membrane in the absence of receptor internalization.

## 2. Materials and Methods

### 2.1. Tissue Culture

BHK-570 cells (ATCC) were cultured as a tissue monolayer using DMEM media (CellGro) containing 10% fetal bovine serum (Atlanta Biological) without antibiotics. Cells were fed every four days and passaged when they were confluent. Culture conditions were set at 37°C, 5% CO_2_, and 80% humidity.

### 2.2. Plasmids

Plasmid DNA encoding MCHR1 in pcDNA3 was purchased from the Missouri S&T cDNA Resource Center. pcDNA3.1+ plasmid encoding MCHR1-VSVg was obtained from Dr. G. Milligan at the University of Glasgow. Dr. J. Benovic at Thomas Jefferson University generously provided plasmids encoding *β*-arrestin 1 and *β*-arrestin 2 in pEGFP-N as well as GRK2 and GRK2 K220R in pRK5. The leptin promoter-driven luciferase reporter plasmid p(-762)ob luc was a gift from Dr. O. Gavrilova at the NIH.

### 2.3. Transfection

Cell transfections were performed on cells plated for at least 24 h with LipoD293 reagent following the recommended protocol from SignaGen. Media were changed 5 hours after transfection and experiments were run approximately 48 hours after transfection. 

### 2.4. ERK Activation Assay

BHK-570 cells in 35 mm dishes were transfected with 1 *μ*g MCHR1 in pcDNA3 or empty plasmid on day 1. On day 2, cells were serum starved in DMEM for 18–24 hours. On the third day, baseline ERK activation was obtained by treating cells for up to 30 min with 1 *μ*M MCH (rat, American Peptide) prior to harvesting in 2X Laemmli sample buffer. Desensitization assays were performed by treating cells with a desensitizing dose of MCH for 15 min followed by rinsing with and incubation of cells in DMEM in the incubator for 30 min (called washout). To activate the pathway again, cells were treated with a second dose of MCH for up to 30 min prior to harvesting in 2X Laemmli sample buffer. Lysates were boiled for 5 min and spun at top speed in a microcentrifuge prior to SDS-PAGE. Proteins were transferred to nitrocellulose and western blots performed with antibodies to both phosphorylated and total ERK (Cell Signaling). Bands were exposed using Western Lightning Enhanced Chemiluminescence kit (Perkin Elmer) on Kodak film.

### 2.5. Fixed Cell Fluorescence Microscopy

Coverslips seeded with BHK-570 cells were transfected with MCHR1-VSVg ± GFP-tagged *β*-arrestin 1 or 2. Cells were then incubated for approximately 24 h and then treated with ±1 *μ*M MCH (American Peptide) for up to 30 min. The coverslips were then rinsed twice with ice-cold PBS before fixing with 4% paraformaldehyde in PBS for 10 min at room temperature. After 3 more washes with PBS, they were transferred to a humidification chamber for incubation in blocking buffer (PBS, 5% goat serum, 0.1% Triton X-100) for at least 20 min. They were then incubated for at least 1 h with rabbit anti-VSVg polyclonal antibody (Sigma) at 1 : 50 in blocking buffer. Following 3 PBS washes, DAPI (Roche) was added at 1 : 500 and Alexa Fluor 546 goat anti-rabbit secondary antibody (Invitrogen) at 1 : 250 in blocking buffer for 1 h. After 3 more PBS washes, coverslips were mounted on glass slides with Prolong Gold (Invitrogen). Fluorescence micrographs were obtained with a Zeiss Axioskop fluorescence microscope with a Zeiss Axiocam camera and Zeiss Axiovision software. Image cropping was performed in Adobe Photoshop and figures assembled in MS PowerPoint. For Figure 4, overlay composite color images were processed in NIH ImageJ to extract individual colors.

### 2.6. Cell-Based ELISA

Twenty-four well plates were seeded with BHK-570 cells and transfected with MCHR1-VSVg ± GFP *β*-arrestin 1 or 2 or in a separate experiment GRK2 or K220R GRK2. Twenty-four hours after transfection, the cells were incubated in labeling buffer (DMEM, 5% goat serum, 0.02 mM HEPES) with 1 : 1,000 mouse anti-VSVg (Sigma) for 2 h. The cells were then washed twice in labeling buffer before being treated with 1 *μ*M MCH for up to 30 min. The cells were then washed once with ice cold PBS and fixed with 3% paraformaldehyde in PBS for 20 min at room temperature. Following 2, 5 min washes with PBS, cells were incubated with goat anti-mouse HRP-conjugated secondary antibody (Bio-Rad) in PBS with 5% goat serum for 45 minutes. After another 3 PBS washes, 175 *μ*L of POD blue (Roche) was added to develop for 3 min on an orbital shaker. The reaction was neutralized with 175 *μ*L of 10% sulfuric acid. One hundred fifty microliters from each well were transferred to a 96-well plate and the absorbance was read at 450 nm using a Synergy 1 or ELx800 plate reader (BioTek).

### 2.7. Leptin Promoter Activation Assay

BHK-570 cells were triple transfected with either (1) MCHR1/p(-762)ob luc/GFP in pcDNA3, (2) MCHR1/p(-762)ob luc/GFP-*β*-arrestin 1, or (3) MCHR1/p(-762)ob luc/GFP *β*-arrestin 2. The cells were treated 48 h after transfection with 1 *μ*M MCH for up to 6 h in DMEM and then lysed and luminol was added. Light production was measured over 15 sec on a BioTek Synergy H1 plate reader. 

### 2.8. Statistical Analyses

The averages of the data were reported ± the standard error of the mean (SEM). Student's *t*-test was performed to determine statistical significance with data scoring equal to or greater than the 95th percentile considered significant. 

## 3. Results

### 3.1. MCH-Mediated Desensitization of ERK Signaling

**Figure 1:**
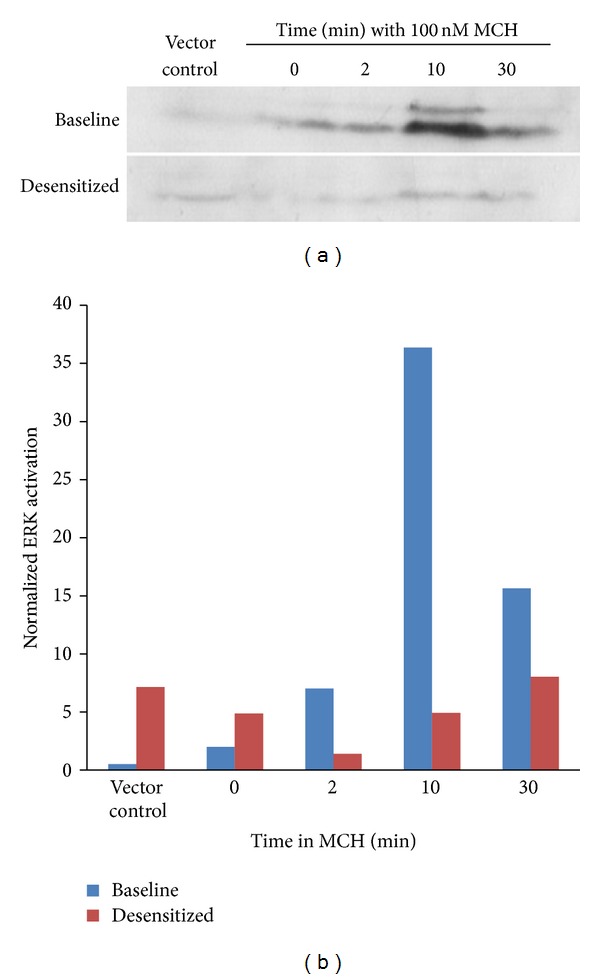
MCH-mediated ERK activation efficiently desensitizes. BHK-570 cells were transfected with plasmid DNA encoding MCHR1 an ERK activation assay performed on cells treated with MCH for up to 30 min. A second set of dishes was treated for MCH for 15 min and then subjected to a 30 min washout prior to the MCH time course (2nd exposure). (a) Cells were harvested in 2X Laemmli sample buffer, run on SDS-PAGE and western blots were performed with primary antibodies to phosphorylated and total ERK. (b) Densitometry was performed using NIH Image J Software where ERK activation was normalized against total ERK. Experiment shown is representative of three total experiments.

It was previously reported that MCH caused activation of the MAPK pathway and leptin promoter in 3T3-L1 adipocytes and that MCHR1 was subsequently downregulated in these cells [12]. To see if MCHR1 could signal to ERK in our model system, we transfected BHK-570 cells with MCHR1 and stimulated them with MCH for 0, 2, 10, and 30 min prior to lysis. Western blots were performed using antibody directed towards phosphorylated and total ERK. We observed transient ERK activation in response to MCH that was not detectable in empty vector transfected control cells as seen in Figure 1(a) as baseline activation, which is quantitated in Figure 1(b) by normalizing activated ERK to total ERK (blots not shown). Within 10 min we measured a greater than 30-fold activation of ERK over baseline that disappeared by 30 min. Surprisingly, when hormone was removed, cells were washed extensively and allowed to recover for up to 30 min and MCH did not regain its ability to activate ERK, indicating significant desensitization of the pathway (Figures 1(a) and 1(b)). We previously showed that an N-terminally tagged VSVg-MCHR1 construct efficiently activates ERK in response to MCH binding [13], and preliminary desensitization experiments conducted with this construct in CHO-K1 cells had similar results to those in Figure 1. Therefore, we conclude that the presence of the VSVg tag does not interfere with receptor signaling (data not shown), and we took advantage of the sensitivity of this epitope to explore the role that receptor internalization plays in controlling the duration of MCH-mediated ERK signaling.

### 3.2. A Small Percentage of MCHR1 Internalizes in Response to MCH

**Figure 2:**
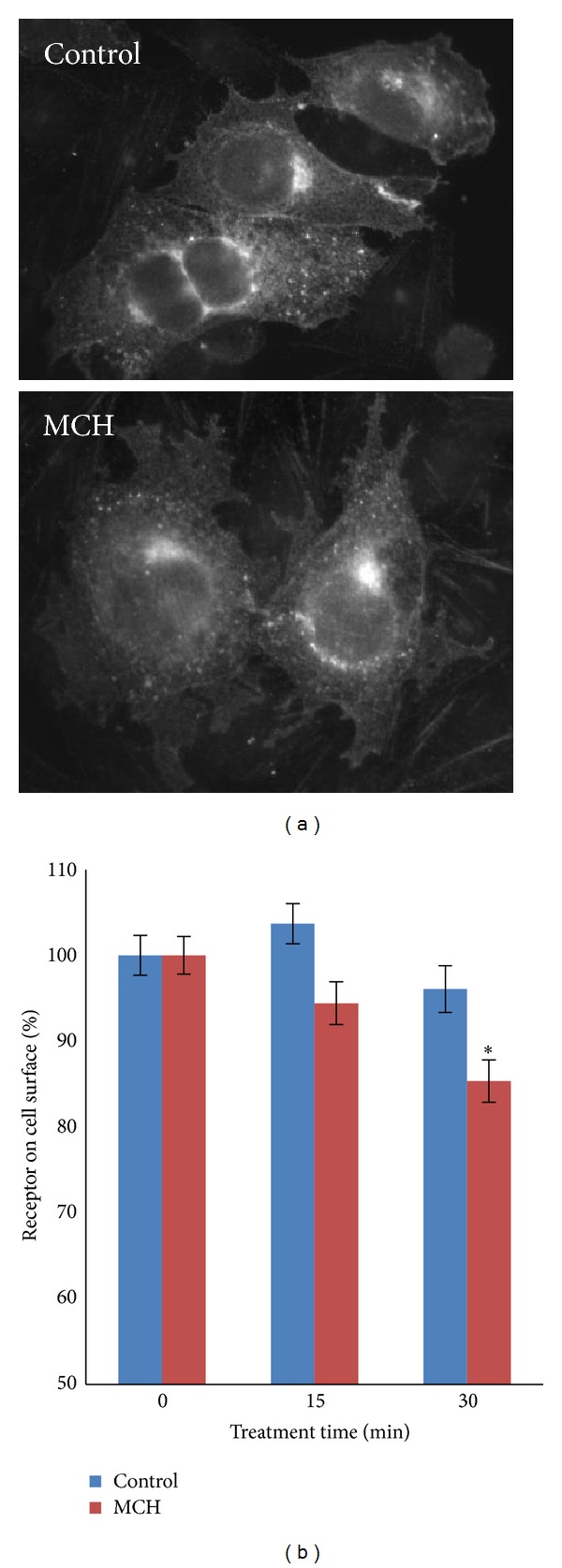
MCH initiates the removal of some MCHR1 from the plasma membrane. BHK-570 cells were transfected with VSVg-tagged MCHR1. (a) Untreated cells and cells that were exposed to 1 *μ*M MCH for 30 min were fixed and immunostained with mouse anti-VSVg primary antibody and goat anti-mouse Alexa Fluor 488 secondary antibody. (b) The loss of MCHR1 from the plasma membrane in response to 1 *μ*M MCH was measured using a modified cell-based ELISA protocol. Background signal from mock-transfected cells was subtracted. *n* = 18 (6 triplicate experiments). *denotes statistical significance at *P* < 0.0025 when compared to 100% control using Student's *t*-test.

The desensitization of MCHR1 was first described by Lembo et al., who observed internalization of MCHR1 through fluorescence microscopy following agonist treatment [9]. We hypothesized that internalization of MCHR1 could be responsible for the extensive desensitization of the ERK signaling pathway by MCH. Much to our surprise, we have been unable to observe any agonist-induced redistribution of MCHR1 using VSVg-tagged MCHR1 expressed in BHK-570 cells by fluorescence microscopy (Figure 2(a)). This was neither specific to the construct (MCHR1-eYFP did not internalize) nor was it specific to BHK-570 cells (both CHO-K1 cells and 3T3-L1 cells failed to internalize MCHR1) (data not shown). We thought that perhaps our method was not sensitive enough to capture the receptors being internalized so we decided to utilize a modified cell-surface ELISA [14] to measure agonist-mediated internalization of an N-terminal VSVg-tagged MCHR1. The VSVg-tag allowed us to utilize a high-affinity antibody for this assay. We measured surface-localized receptor in untreated cells (100% ± 2.3%), and compared to cells treated with MCH for 15 or 30 min, the results of which are shown in Figure 2(b). It was quite surprising that MCH was only able to drive surface receptor levels after 15 min to 96% ± 2.7% of control and after 30 min to 85% ± 2.5% (*P* < 0.0025) of control, which was confirmed by fluorescence microscopy (Figure 2(a)). To determine if high expression levels of VSVg-MCHR1 contributed to the low internalization levels, we titrated back plasmid DNA amounts during transfection and found that it did not improve internalization (data not shown). This low level of internalization was also not a consequence of the N-terminal tag because Murdoch and colleagues were able to observe internalization of this construct in HEK 293 cells by fluorescence microscopy [15]. Others utilized flow cytometry to measure MCH-mediated removal of MCHR1 from the plasma membrane [10, 11].

### 3.3. Overexpression of *β*-Arrestins Rescues MCH-Mediated MCHR1 Internalization

**Figure 3:**
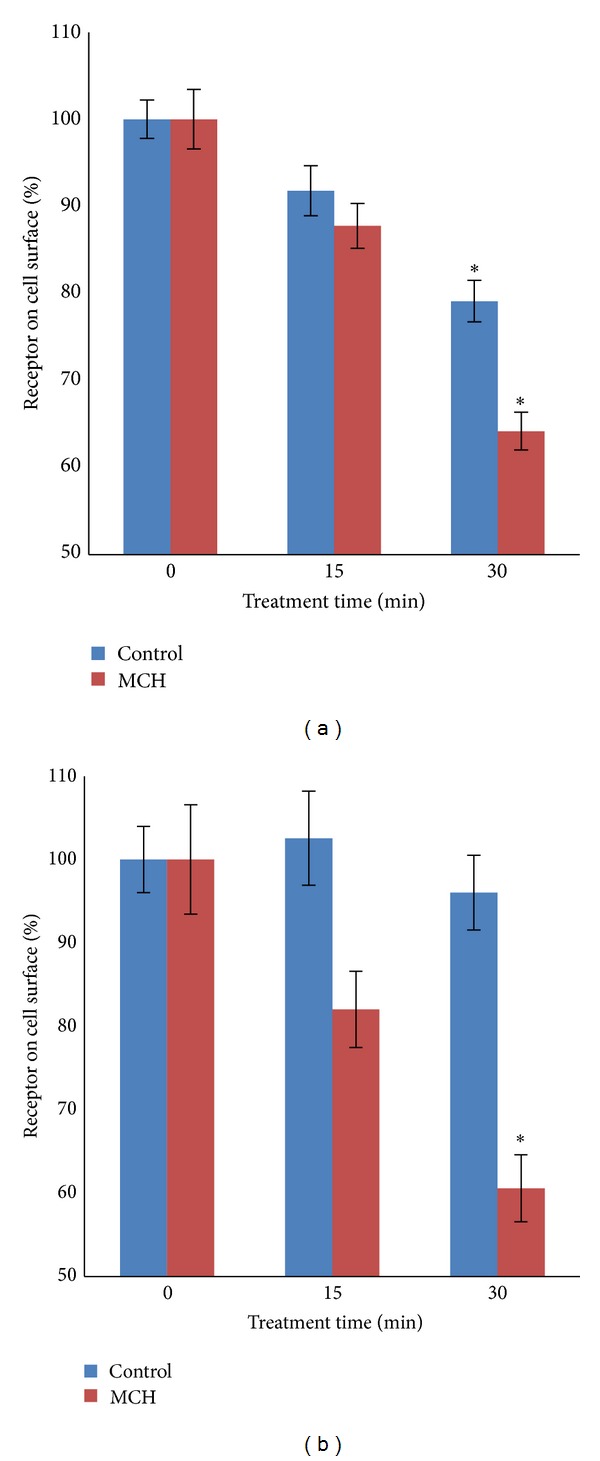
Coexpression of MCHR1 with *β*-arrestin 1 or 2 enhances MCH-mediated receptor internalization. BHK cells were cotransfected with 1 *μ*g each plasmids encoding VSVg-MCHR1 and (a) GFP *β*-arrestin 1 or (b) GFP *β*-arrestin 2 or GFP control plasmid (both (a) and (b)). The loss of MCHR1 from the plasma membrane in response to 1 *μ*M MCH was measured using a modified cell-based ELISA protocol. Background signal from mock-transfected cells was subtracted. For *β*-arrestin 1, *n* = 21 (7 triplicate experiments); for *β*-arrestin 2, *n* = 15 (5 triplicate experiments). Ave ± SEM plotted. *P < 0.01.

**Figure 4:**
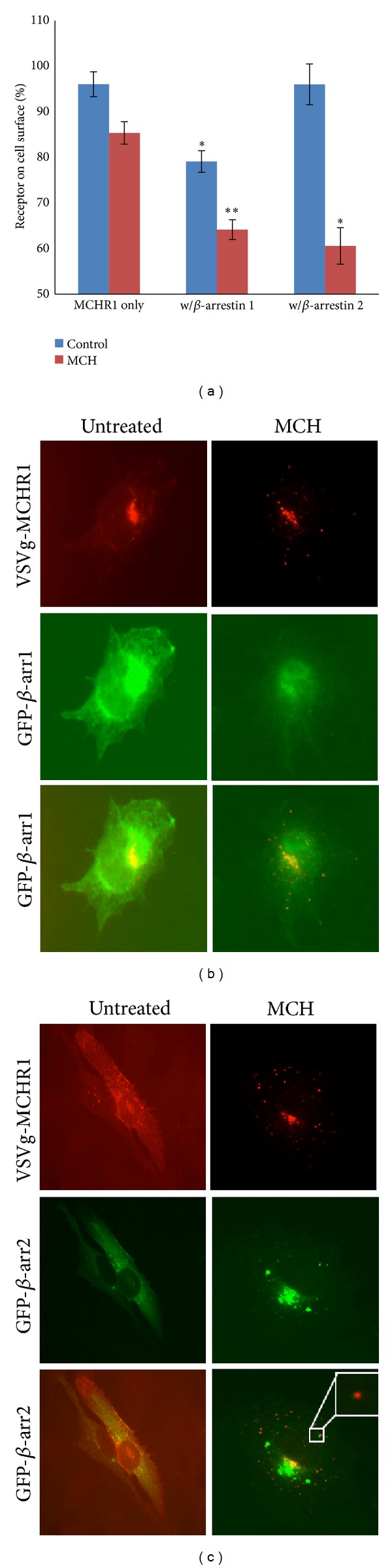
Overexpression of *β*-arrestins differentially influences MCHR1 internalization. BHK-570 cells were transfected with VSVg-MCHR1 and either *β* arrestin-1 or *β* arrestin-2. (a) Cells were treated with 1 *μ*M MCH or vehicle for 30 min and internalization was measured utilizing a cell-based ELISA. Data from Figure 3 was replotted. Ave ± SEM plotted. **P < 0.005, *P < 0.01. VSVg-MCHR1 and GFP *β*-arrestin 1-expressing cells (b) and VSVg-MCHR1 and GFP *β*-arrestin 2-expressing cells (c) were treated with 1 *μ*M MCH or vehicle for 30 min, then fixed, and immunostained with anti-VSVg antibody. Fluorescence microscopy was performed and overlay images compared.

We wondered whether MCHR1 could interact with the machinery to internalize in our model system, and so we decided to coexpress VSVg-MCHR1 with either *β*-arrestin 1 or *β*-arrestin 2 and again measure internalization. Overexpression of *β*-arrestins has been shown to increase the internalization of *β*_2_-adrenergic receptors [16] and it was hypothesized that *β*-arrestin would increase the rate of MCHR1 internalization in response to MCH. When *β*-arrestin 1 was overexpressed, surface-localized MCHR1 unexpectedly declined in the absence of agonist from 100% ± 3.4% to 92% ± 2.6% at 15 min and 79% ± 2.2% (*P* < 0.01) at 30 min. Treatment with MCH however further improved the removal of MCHR1 from the cell surface to 88% ± 2.9% of the total at 15 min and only 64% ± 2.4% after 30 minutes (Figure 3(a)). When this experiment was repeated with *β*-arrestin 2, in contrast to *β*-arrestin 1 we found steady surface levels of MCHR1 over the course of 30 min in the absence of agonist. When cells were treated with hormone for 15 min, surface receptor levels fell from 100% ± 6.5% to 83% ± 4.6% and by 30 min declined to 62% ± 4.0% (*P* < 0.01) (Figure 3(b)). We directly compared the effects that each *β*-arrestin has on loss of MCHR1 from the cell surface of BHK-570 cells treated with MCH for 30 min in Figure 4. In our most successful internalization experiments, 60% of the receptor still remained on the plasma membrane despite vast overexpression of arrestins (data not shown) and supraphysiological agonist concentrations. Coexpression of MCHR1 with *β*-arrestin 1 resulted in an agonist-independent net loss of 17.0% of MCHR1 from the plasma membrane and an agonist-dependent net loss of 21.1%. The coexpression of *β*-arrestin 2 resulted in no appreciable agonist-independent net loss and an agonist-dependent net loss of 24.7% (*P* < 0.01). 

**Figure 5:**
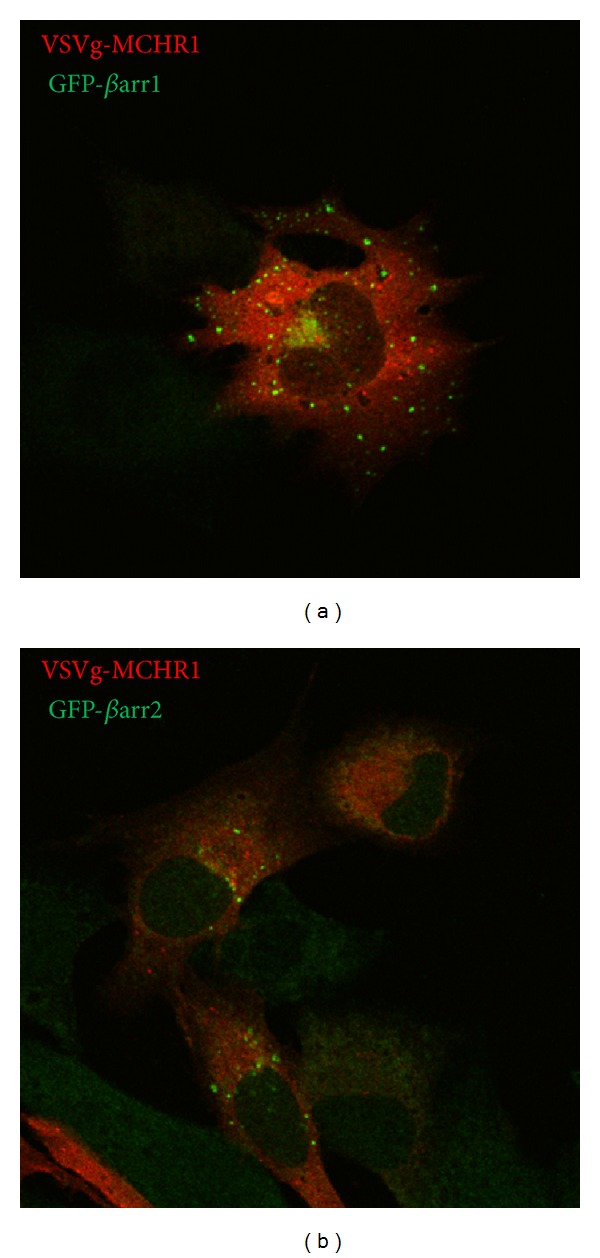
VSVg-MCHR1-containing vesicles are distinct from those associated with *β*-arrestins. VSVg-MCHR1 and GFP *β*-arrestin 1-expressing cells (a) and VSVg-MCHR1 and GFP *β*-arrestin 2-expressing cells (b) were treated with 1 *μ*M MCH for 30 min, then fixed, and immunostained with anti-VSVg antibody. Confocal microscopy was performed using a grid pattern confocal microscope and Image-Pro Plus software. A single slice is shown as an overlay of both Alexa Fluor (546 nm) and GFP (488 nm) signals.

These experiments highlight some differences between the association amongst the receptor and each arrestin; while both arrestins facilitated coupling of MCHR1 with the internalization machinery, *β*-arrestin 1 seems to have facilitated receptor coupling in the absence of agonist. To verify this visually, fluorescence microscopy was performed on fixed cells. *β*-arrestin 1 overexpression seems to result in a large amount of both receptor and arrestin to be stuck in a juxtanuclear compartment (Figure 4(b)). This could either be the result of inefficient trafficking of newly synthesized receptor to the plasma membrane or excessive removal of unoccupied receptors from the plasma membrane. Our ELISA data supports the latter hypothesis (Figures 3 and 4). Although some *β*-arrestin 1 was not visibly recruited to the plasma membrane in these cells (Figure 4(b), middle panel), treatment of cells with MCH for 30 min did show MCHR1 accumulation in punctate vesicles (Figure 4(b), top panel); these vesicles lacked *β*-arrestin 1 (Figure 4(b), bottom panel). *β*-arrestin 2 gave a similar, but more extensive internalization profile with a larger number of MCHR1-positive puncta (Figure 4(c), top panel) that are also positive for GFP-*β*-arrestin 2 when the overlay is examined (Figure 4(c) middle and bottom panels). This data agrees with Evans and colleagues by demonstrating that the MCHR1-*β*-arrestin 2 interaction is of higher affinity for the receptor than that of *β*-arrestin 1 [10]. To further explore the potential colocalization of VSVg-MCHR1 with both *β*-arrestins, confocal microscopy was performed. Shown in Figure 5 is a representative single plane where colocalization of neither *β*-arrestin with VSVg-MCHR1 is readily apparent after a 30 min hormone treatment. Instead, most MCH receptor-containing vesicles are distinctly separate from *β*-arrestin-associated vesicles. Thus the apparent colocalization observed in Figure 4 could not be verified. Future experiments should explore this biochemically.

### 3.4. GRK2 Plays a Role in MCHR1 Internalization

**Figure 6:**
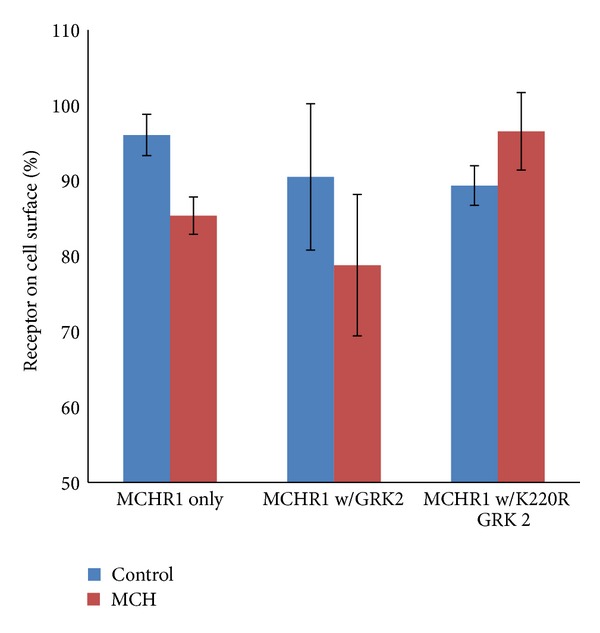
Perturbations of cellular GRK2 levels affect MCH-mediated internalization of MCHR1. BHK570 cells were transfected with VSVg-MCHR1 and either GRK2 or K220R GRK2. Cells were treated with 1 *μ*M MCH or vehicle for 30 min and internalization was measured utilizing a cell-based ELISA. *n* = 18 for control; *n* = 15 for GRK2; *n* = 9 for K220R GRK2. Ave ± SEM plotted. Experiment reached the 90% confidence interval but did not reach statistical significance.

G protein-coupled receptor kinase 2 (GRK2) is a family member of a group of protein kinases that generally function to regulate GPCR signaling through the phosphorylation of serine and threonine residues located on the intracellular loops and C-terminal tails of GPCRs. The addition of phosphate groups to these areas has been shown to terminate signaling by inhibiting G protein coupling and facilitating receptor internalization by increasing the affinity of arrestins for the receptor [17]. GRK2 plays a role in both *β*-arrestin-dependent and *β*-arrestin-independent trafficking of GPCRs [18]. A dominant-negative form of GRK2, GRK2 K220R has already been characterized [19] and is known to knock down the expression of wild-type GRK2 when coexpressed. Based on previous studies [20], it was hypothesized that the overexpression of GRK2 would increase the rate of internalization, while transfection of the K220R GRK2 would knock down endogenous GRK2 resulting in inhibited MCHR1 internalization. MCHR1 internalization was monitored by cell-surface ELISA over the course of 30 min. Cotransfection of MCHR1 with either GRK2 or K220R GRK2 resulted in no appreciable loss of receptors in the absence of agonist (Figure 6). An agonist-dependent loss of receptors equating to 6.5% (*P* < 0.005) for GRK2-expressing cells was measured and a net increase in surface receptor levels of 11.3% (*P* < 0.005) for K220R-expressing cells was measured when compared to cells given a control plasmid.

### 3.5. MCH Signaling to a Leptin Promoter Is Attenuated by Coexpression of Arrestins

**Figure 7:**
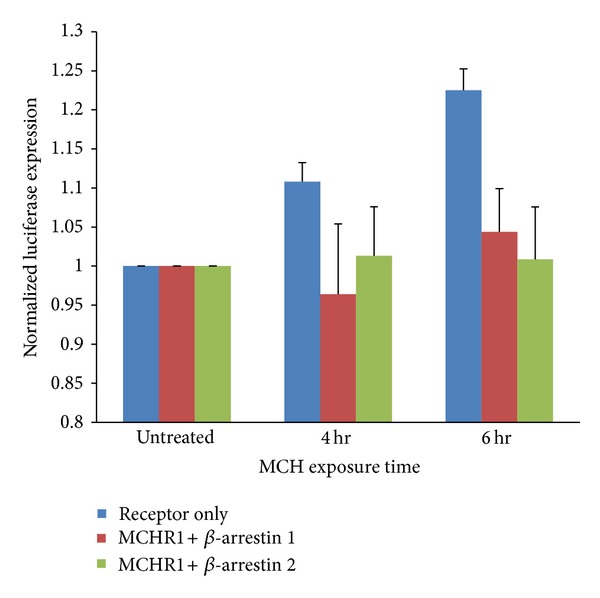
*β*-arrestins diminish the activation of a leptin promoter by MCH. BHK 570 cells triple transfected with either (1) MCHR1/p(-762)ob luc/GFP in pcDNA3, (2) MCHR1/p(-762)ob luc/GFP-*β*-arrestin 1, or (3) MCHR1/p(-762)ob luc/GFP-*β*-arrestin 2 were treated with 1 *μ*M MCH for up to 6 h. A luciferase assay was then performed to quantifiably measure the activation of the leptin promoter. *n* = 5 for control and *n* = 3 for each *β*-arrestin experiment. Graph shows averages of normalized data ± SEM. Experiment reached the 85% (*β*-arr2) and 90% (*β*-arr1) confidence intervals but did not reach statistical significance.

In order to determine if this loss of receptor from the cell surface translates into improved desensitization of MCH signaling, we utilized a reporter plasmid from which luciferase expression is driven by activation of the leptin promoter. This construct was previously reported to respond to MCH over the course of several hours [21]. As shown in Figure 7, cells expressing *β*-arrestin 1 or 2 showed less luciferase expression compared to control in response to MCH treatment for up to 6 h. This indicates that MCH-mediated activation of the leptin promoter can be desensitized by overexpression of arrestins and it supports the hypothesis that agonist-mediated internalization of MCHR1 acts to desensitize cells to MCH.

## 4. Discussion

Melanin-concentrating hormone acts on at least two G protein-coupled receptors in the central nervous system and peripheral organs to elucidate a variety of physiological responses. A few early studies suggested that at least one of those receptors, melanin-concentrating hormone receptor 1, undergoes phosphorylation, *β*-arrestin 2 recruitment, and agonist-mediated internalization [9–11]. However, when we attempted to replicate these experiments much to our surprise we were unable to measure internalization of MCHR1 visually (Figure 2(a)) and when measured quantitatively, only weak (~15%) internalization was observed (Figures 2(b) and 3) despite extensive desensitization of the ERK pathway (Figure 1). Our observations were not limited to BHK-570 cells, but MCHR1 internalization was also not observed in CHO-K1 cells or 3T3-L1 cells for various receptor constructs including MCHR1-eYFP (data not shown). This is in contrast to results described by others who observed internalization of MCHR1 by other means [9–11]. The clathrin-mediated endocytosis pathway has been widely characterized from studies of other GPCRs [22]. *β*-arrestins are important mediators, helping to recruit clathrin heavy chain to GPCRs, and receptors such as the *β*_2_-adrenergic receptor are known to desensitize through this pathway [21–23]. 

The relative levels of GPCR, *β*-arrestin, and GRK have been previously implicated in sequestration efficiency as reported by Barak and colleagues for the *β*_2_-adrenergic receptor [24]; however, these differences probably do not entirely explain the results we see with MCHR1. mGlu1R*β* [25] and GHRH-R [26] are two GPCRs that when heterologously expressed in BHK-570 cells internalized rapidly following agonist exposure, suggesting that baby hamster kidney fibroblast cells indeed have the capacity to sequester GPCRs. When we coexpressed *β*-arrestins 1 and 2 or GRK2 with MCHR1, we were able to partially rescue internalization of the receptor (Figures 3-4). Our results suggest that *β*-arrestin 1 is indeed capable of coupling MCHR1 to the endocytic machinery, but that the association is weak because cointernalization of the arrestin with the receptor was not observed (Figure 4(b)). We also presented evidence of agonist-independent removal of MCHR1 from the cell surface with *β*-arrestin 1 in Figures 3(a) and 4(a), although this could be the result of an antibody-induced conformational change that results in internalization of the receptor. When *β*-arrestin 2 was coexpressed, co-internalization with MCHR1 following agonist treatment was observed (Figure 4(c)), but not confirmed with confocal microscopy methods (Figure 5). These results mirror those from a study done with *β*_2_ adrenergic receptor in which cells triple transfected with *β*_2_-adrenergic receptor, *β*_2_-adrenergic receptor kinase, and *β*-arrestins showed a significant synergistic effect [24]. 

The suggestion that MCHR1 favors *β*-arrestin 2 is not a new one. Evans et al. previously reported selective, but transient recruitment of *β*-arrestin 2 to the plasma membrane of transfected HEK293 cells following MCH exposure [10]. Unlike their study, in our experimental system utilizing BHK-570 cells it was not necessary to overexpress GRK2 to observe either *β*-arrestins' effect on MCHR1 internalization. Also, although we did not specifically detect recruitment of the GFP *β*-arrestins to the cell surface, it is implied since their presence efficiently facilitated coalescing of receptors into punctate vesicles for internalization. Saito and colleagues, using HEK293T cells, overexpressed *β*-arrestin 2 and showed no significant effects on MCH-mediated receptor internalization. However, a dominant-negative version seemed to inhibit it [11]. These seemingly conflicting results may have been the result of inefficient GRK2 levels to accompany the overexpressed *β*-arrestin 2. The best illustration of cell-to-cell variation in internalization efficiency is for the *β*_2_-adrenergic receptor [24], and abnormalities in GRK or *β*-arrestin protein levels have been linked to Alzheimer's disease [27], inflammatory diseases [28], and cardiac ailments [17]. Our results illustrate that protein levels of both GRK2 and *β*-arrestin have the potential to modulate the response of cells to MCH, potentially influencing human appetite.

G protein-coupled receptors can be divided into two classes: Class A receptors and Class B receptors. Class A receptors preferentially bind *β*-arrestin 2 over *β*-arrestin 1 and their interaction is transient in nature with *β*-arrestin and the receptor dissociating prior to entering the endosome. Class B receptors have equal affinity for both *β*-arrestin 1 and *β*-arrestin 2 and form stable interactions resulting in the translocation of both receptor and *β*-arrestin into the endosome [29]. When both *β*-arrestins were coexpressed with MCHR1 in this study, receptor internalization was facilitated significantly over control, but to a greater extent with *β*-arrestin 2, suggesting that MCHR1 is a Class A receptor. If cointernalization of *β*-arrestin 2 with MCHR1 really does occur in our model system, it would suggest that MCHR1 is a Class B receptor. MCHR1 may have an intermediate phenotype in BHK-570 cells. 

The protein kinase GRK2 is known to phosphorylate serine threonine residues on the third intracellular loop and C-terminal tail of GPCRs increasing their affinity for *β*-arrestins in an agonist-dependant manner [17]. Up until now, the effect of GRK2 overexpression on the desensitization of MCHR1 had yet to be explicitly measured although, as already stated, Evans et al. utilized GRK2 transfected cells while observing the nature of the interaction of *β*-arrestins with MCHR1 [10]. Agonist-induced internalization of MCHR1 while overexpressing dominant-negative GRK 2 showed no significant increase in the rate of receptor internalization (Figure 5) similar to the *μ*-opioid receptors. These receptors are dependent on phosphorylation by GRKs for the recruitment of *β*-arrestins in contrast to that of the *β*_2_ adrenergic receptor, which can interact with *β*-arrestins in the absence of GRK2 phosphorylation sites [23, 24]. This suggests that MCHR1 is dependent on GRK2 phosphorylation for the recruitment of *β*-arrestins and together with previous data and that the rate-limiting step in MCHR1 desensitization is dependent on the cellular concentration of *β*-arrestins and/or GRK2. This would be true, however, if internalization was required for desensitization of MCH signaling. Considering that only 15% of receptors internalized in our model system (Figure 2) yet ERK signaling was efficiently desensitized (Figure 1), we hypothesize that desensitization of the MCH signaling pathway in these cells does not solely rely on removal of receptors from the plasma membrane.

We hypothesized that overexpression of arrestins would not only promote agonist-mediated internalization of MCHR1, but that this would indeed translate into a desensitization of downstream signaling. Our results in Figure 7 support a role for *β*-arrestins in downregulating this pathway because MCH-mediated luciferase accumulation is essentially absent when they are coexpressed with MCHR1. This suggests that MCH-mediated ERK signaling is spatially linked to membrane-localized MCHR1 and that agonist-mediated internalization of MCHR1 is not necessary for ERK signaling, rather it may participate in signal termination.

MCHR1 signaling to G proteins is thought to be regulated differentially by at least two RGS proteins, RGS2 and RGS8 [25, 26]. These proteins act to promote GTP hydrolysis inactivating the G protein signal. MCH-mediated ERK signaling in HEK293 cells proceeds via G*α*q and G*α*i [30], both of which are targets of RGS8 [25, 26]. It is not known whether RGS8 plays a role in desensitizing ERK signaling in BHK-570 cells, but it seems a likely candidate.

Another likely possibility is that MCHR1 becomes spatially segregated from its signaling components in the plasma membrane. MCHR1 forms a complex with caveolin-1 in these cells, and MCHR1 is highly enriched in caveolae [13]. It has been reported that G*α*i and G*α*s are able to migrate into and out of caveolae unlike G*α*q, which is tethered there [31]. Interestingly, adipocytes express exceedingly high levels of caveolin-1; therefore regulation of MCH signaling via caveolae or other lipid rafts in this cell type seems to be a strong possibility. The obese phenotype seen as a result of primary cilia loss in Bardet-Biedl syndrome is hypothesized to be the result of a loss of cilia-localized MCHR1 in the brain [7]. Since MCH signals appetite, this suggests that ciliary localization of MCHR1 dampens the MCH signal, further evidence to suggest that spatial organization of these receptors in the plasma membrane contributes to the regulation of its activity.

## 5. Conclusion

ERK signaling by MCH potently desensitizes in BHK-570 cells. We tested the hypothesis that agonist-induced removal of MCH receptors from the plasma membrane was largely responsible for this process. Surprisingly we found that MCH receptors internalize very poorly in BHK-570 cells unless *β*-arrestin 1 or *β*-arrestin 2 is overexpressed. Similarly, GRK2 phosphorylation of MCHR1 is thought to be important because a dominant-negative GRK2 was able to eliminate even the small amount of receptor internalization induced by MCH. We conclude that MCH receptor signaling and desensitization are particularly sensitive to cellular levels of *β*-arrestins and GRK2, which should be strongly taken under consideration when interpreting MCH signaling studies across different cell types. Perturbation of the cellular levels of these accessory proteins could greatly influence the activity of this appetite-stimulating pathway.
